# Impact of oxidative stress defense on bacterial survival and morphological change in *Campylobacter jejuni* under aerobic conditions

**DOI:** 10.3389/fmicb.2015.00295

**Published:** 2015-04-10

**Authors:** Euna Oh, Lynn McMullen, Byeonghwa Jeon

**Affiliations:** ^1^School of Public Health, University of Alberta, Edmonton, ABCanada; ^2^Department of Agricultural, Food and Nutritional Science, University of Alberta, Edmonton, ABCanada

**Keywords:** *Campylobacter*, oxidative stress defense, VBNC

## Abstract

*Campylobacter jejuni*, a microaerophilic foodborne pathogen, inescapably faces high oxygen tension during its transmission to humans. Thus, the ability of *C. jejuni* to survive under oxygen-rich conditions may significantly impact *C. jejuni* viability in food and food safety as well. In this study, we investigated the impact of oxidative stress resistance on the survival of *C. jejuni* under aerobic conditions by examining three mutants defective in key antioxidant genes, including *ahpC*, *katA*, and *sodB*. All the three mutants exhibited growth reduction under aerobic conditions compared to the wild-type (WT), and the *ahpC* mutant showed the most significant growth defect. The CFU reduction in the mutants was recovered to the WT level by complementation. Higher levels of reactive oxygen species were accumulated in *C. jejuni* under aerobic conditions than microaerobic conditions, and supplementation of culture media with an antioxidant recovered the growth of *C. jejuni*. The levels of lipid peroxidation and protein oxidation were significantly increased in the mutants compared to WT. Additionally, the mutants exhibited different morphological changes under aerobic conditions. The *ahpC* and *katA* mutants developed coccoid morphology by aeration, whereas the *sodB* mutant established elongated cellular morphology. Compared to microaerobic conditions, interestingly, aerobic culture conditions substantially induced the formation of coccoidal cells, and antioxidant treatment reduced the emergence of coccoid forms under aerobic conditions. The ATP concentrations and PMA–qPCR analysis supported that oxidative stress is a factor that induces the development of a viable-but-non-culturable state in *C. jejuni*. The findings in this study clearly demonstrated that oxidative stress resistance plays an important role in the survival and morphological changes of *C. jejuni* under aerobic conditions.

## Introduction

*Campylobacter jejuni* is one of the leading foodborne pathogens worldwide and is frequently found in the intestines of a wide range of wildlife and domestic animals, particularly poultry (Lee and Newell, 2006; Humphrey et al., 2007). As a microaerophile, *C. jejuni* is sensitive to high oxygen concentrations in the atmosphere (Lee and Newell, 2006). However, ironically, *C. jejuni* is isolated from various environmental sources and foods in normal atmospheric conditions with high oxygen tension (Luber and Bartelt, 2007; Guerin et al., 2010; Trimble et al., 2013). Multiple factors have been reported to affect *C. jejuni* survival in oxygen-rich conditions. For example, pyruvate protects *C. jejuni* from high oxygen stress in aerobic conditions (Verhoeff-Bakkenes et al., 2008), and a combinational use of ferrous sulfate, sodium metabisulfite and sodium pyruvate in culture media increases the viability of *C. jejuni* (Chou et al., 1983). In addition, metabolic commensalism with *Pseudomonas* sp. in spoilage microflora in chicken meat is also known to affect the survival of *C. jejuni* under aerobic conditions (Hilbert et al., 2010).

Since oxygen easily moves across the membrane, oxygen concentrations within a cell are equivalent to those of immediate extracellular environments (Imlay, 2008). Bacterial growth in the presence of oxygen inevitably produces reactive oxygen species (ROS) that may damage intracellular macromolecules, such as DNA, proteins, and lipids; thus, bacteria are equipped with a range of oxidative stress defense systems to detoxify ROS (Storz and Imlay, 1999; Imlay, 2008). To reduce exposure to high oxygen tension, unlike aerobes, microaerophiles, and anaerobes live in habitats where oxygen levels are low (Imlay, 2008), but conserved oxidative stress resistance systems are still available in microaerophilic bacteria and even in obligate anaerobes (Chiang and Schellhorn, 2012). The most common ROS-detoxification enzymes would include alkyl hydroperoxide reductase, catalase, and superoxide dismutase (SOD; Imlay, 2008); alkyl hydroperoxide reductase converts organic peroxides to alcohols and detoxifies physiological levels of hydrogen peroxide (Seaver and Imlay, 2001; Parsonage et al., 2008), catalase decomposes hydrogen peroxide to water and oxygen, and SOD dismutates superoxide to hydrogen peroxide and oxygen (Imlay, 2008). Bacteria often possess redundant forms of the ROS-detoxification enzymes. For example, three types of SOD (SodA, SodB, and SodC), and two catalases (KatG and KatE) are present in *Escherichia coli* (Storz and Imlay, 1999; Imlay, 2008). However, the *C. jejuni* genome harbors only single gene copies of *sodB*, *katA*, and *ahpC* (Parkhill et al., 2000; Atack and Kelly, 2009). By using three antioxidant mutants (*ahpC*, *katA*, and *sodB*) defective in the production of key ROS-detoxification enzymes in *C. jejuni*, in this study, we demonstrated that oxidative stress defense play an important role in survival and morphological changes in *C. jejuni* under aerobic conditions.

## Materials and Methods

### Bacterial Strains and Culture Conditions

*Campylobacter jejuni* NCTC 11168 (Parkhill et al., 2000) and its isogenic *ahpC*, *katA*, and *sodB* mutants and their complementation strains (Oh and Jeon, 2014) were used in this study. All *C. jejuni* strains were routinely grown on Mueller Hinton (MH) media at 42°C under microaerobic conditions (5% O_2_, 10% CO_2_, 85% N_2_). MH media were supplemented with kanamycin (50 μg ml^-1^) or chloramphenicol (25 μg ml^-1^), where required. For liquid culture of *C. jejuni*, an overnight culture on MH agar was resuspended in MH broth to an OD600 of 0.07, and the bacterial suspension was grown at 42°C microaerobically or aerobically with shaking at 200 rpm.

### Measurement of Total ROS Level

The level of total ROS accumulation was measured by using the fluorescence dye CM-H_2_DCFA (Life Technologies). *C. jejuni* was prepared by growing in MH broth under the culture conditions described above. Samples were taken at predetermined time (0, 4, 8, and 12 h) and washed with PBS. After treatment with 10 μM CM-H_2_DCFA for 30 min at room temperature, fluorescence was measured with FLUOstar Omega (BMG Labtech, Germany). The fluorescence levels were normalized to protein amounts that had been determined with the Bradford assay (Bio-Rad).

### Aerotolerance Test

*Campylobacter jejuni* was grown in MH broth under aerobic conditions as described above. Samples were collected after 0, 4, 8, and 12 h for serial dilution and bacterial counting. Occasionally, *N*-acetyl cysteine (an antioxidant) was added to the cultures to a final concentration of 1 nM.

### Fluorescence Microscopic Analysis

After cultured in MH broth as mentioned above, *C. jejuni* samples (100 μl) were taken at 0 h and after 12 h culture. Samples were washed twice with PBS and stained with the LIVE/DEAD BacLight Bacteria Viability Kit (Life Technologies). Samples were observed with a fluorescence microscope (Carl Zeiss M100, Germany), and image analysis was performed with AxioVision SE64 (Version 4, Carl Zeiss).

### Determination of Protein Oxidation

*Campylobacter jejuni* strains were grown in MH broth for 12 h as described above. Bacterial cells were washed twice with PBS and disrupted with a sonicator (Bioruptor; Diagenode, USA). Disrupted bacterial samples were loaded onto a 15% polyacrylamide gel after adding a sample buffer. Proteins were transferred from the gel to a PVDF membrane, and carbonylated protein groups were detected immunologically using primary anti-DNP antibody (Sigma–Aldrich). Anti-rabbit-peroxidase antibody was used as the secondary antibody (KPL Inc., USA). Primary and secondary antibodies were used at a dilution of 1:1,000, and the blotting results were visualized with a 4CN Peroxidase substrate system (KPL Inc.).

### Lipid Peroxide (LPO) Assay

Lipid peroxide levels were measured with a commercial kit (Cayman Chemical Co., USA) according to the manufacturer’s instructions. Briefly, *C. jejuni* strains were cultured in MH broth for 12 h as described above. The bacterial samples were washed twice and resuspended in PBS buffer. After thorough mix with an equal volume of Extract R, and following addition of ice cold chloroform to each sample, the bottom chloroform layer was collected by centrifugation at 1,500 × *g* for 5 min at 0°C. Chloroform-extracted samples were mixed with chloroform–methanol. The LPO levels were measured by reading the absorbance at 500 nm after mixing with Chromogen. The results were normalized to the total protein amounts in each sample that were determined with the Bradford assay (Bio-Rad).

### Determination of ATP Concentrations

The ATP concentrations were measured with a commercial kit (Sigma–Aldrich) according to the manufacturer’s instructions. *C. jejuni* NCTC 11168 was grown in MH broth for 12 h as described above. Samples were washed twice with PBS and resuspended with the assay buffer. The bacteria samples were reacted with a substrate and ATP enzyme for 3 min at room temperature. The ATP concentrations were determined by measuring luminescence with FLUOstar Omega. The ATP concentrations were normalized to protein contents in the reaction.

### PMA–qPCR

Prior to the assay, bacterial cells were washed twice with PBS and resuspended with 20 μM PMA (Biotium Inc., USA). After exposure to LED light (Biotium Inc.) for 15 min, genomic DNA was extracted by boiling, and the supernatants were used for qPCR. The qPCR assay was performed with 7500 Fast Real Time PCR System (Applied Biosystems, USA). The amplification program consisted of one cycle at 95°C for 5 min and 40 cycles at 95°C for 30 s, 55°C for 30 s, and 72°C for 45 s. The *Cjr01* gene was used as a control to normalize the results (Hwang et al., 2012), and the data were analyzed by using the 2^-ΔΔCt^ method. The primer sequences are described in **Table 1**.

**Table 1:** The primer sequences used in this study.

Primer	Sequence (5′–3′)	Reference
16s rRNA_F	GGATGACACTTTTCGGAGC	Logan et al. (2001)
16s rRNA_R	CATTGTAGCACGTGTGTC	Logan et al. (2001)
Cjr01_F	TCGAACGATGAAGCTTTTAG	Hwang et al. (2012)
Cjr01_R	TTGTCCTCTTGTGTAGGG	Hwang et al. (2012)

## Results and Discussion

### Impact of ROS Accumulation on *C. jejuni* Viability under Aerobic Conditions

**FIGURE 1:**
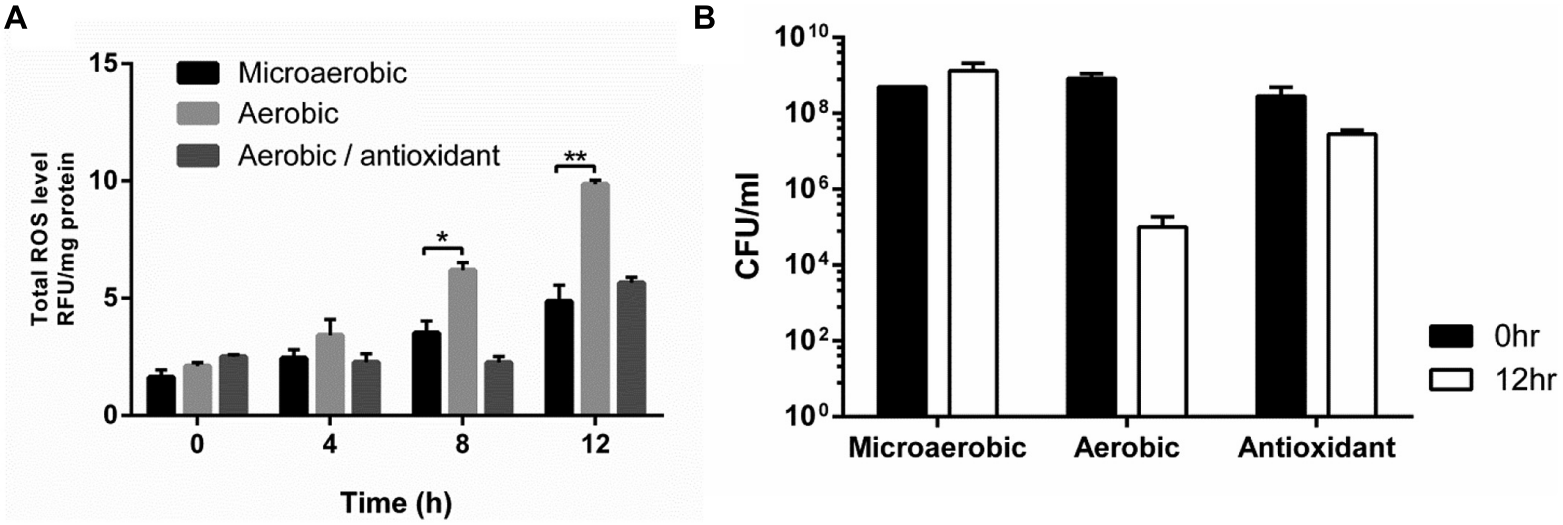
**Total reactive oxygen species (ROS) accumulation **(A)** and *Campylobacter jejuni* viability **(B)** under microaerobic and aerobic conditions**. *N*-acetyl cysteine was used as an antioxidant to a final concentration of 1 nM. The results show the means and SD of triplicate samples in a single experiment. The assays were repeated at least three times, and similar results were observed in all the experiments. Statistical analysis was performed with the Student’s *t*-test using GraphPad Prism 6 (GraphPad Software Inc., USA). *P < 0.05, **P < 0.01.

In contrast to favorable growth conditions available in the gastrointestinal tracts of poultry, such as low oxygen levels, high nutrients, and optimal growth temperatures (i.e., ~42°C), high oxygen tension in the atmosphere is one of the harsh environmental stress that *C. jejuni* should overcome to support its survival during the processing, transport, and storage of poultry products prior to foodborne exposure to humans and establishment of infections. Since ROS is inevitably generated as by-products of bacterial metabolisms in the presence of oxygen, we measured the levels of ROS accumulation in *C. jejuni* under aerobic conditions. The total ROS levels were significantly increased under aerobic conditions compared with microaerobic conditions (**Figure 1A**), and *C. jejuni* viability was substantially decreased (**Figure 1B**). To determine the impact of ROS accumulation on *C. jejuni* viability, the experiment was also performed in the presence of an antioxidant (1 nM *N*-acetyl cysteine). The addition of *N*-acetyl cysteine to aerobic cultures reduced the total ROS levels (**Figure 1A**) and restored *C. jejuni* viability similar to that under microaerobic conditions (**Figure 1B**). These results demonstrate that increased ROS accumulation affects *C. jejuni* viability under aerobic conditions.

### Growth Defects in ROS-Detoxification Mutants under Aerobic Conditions

**FIGURE 2:**
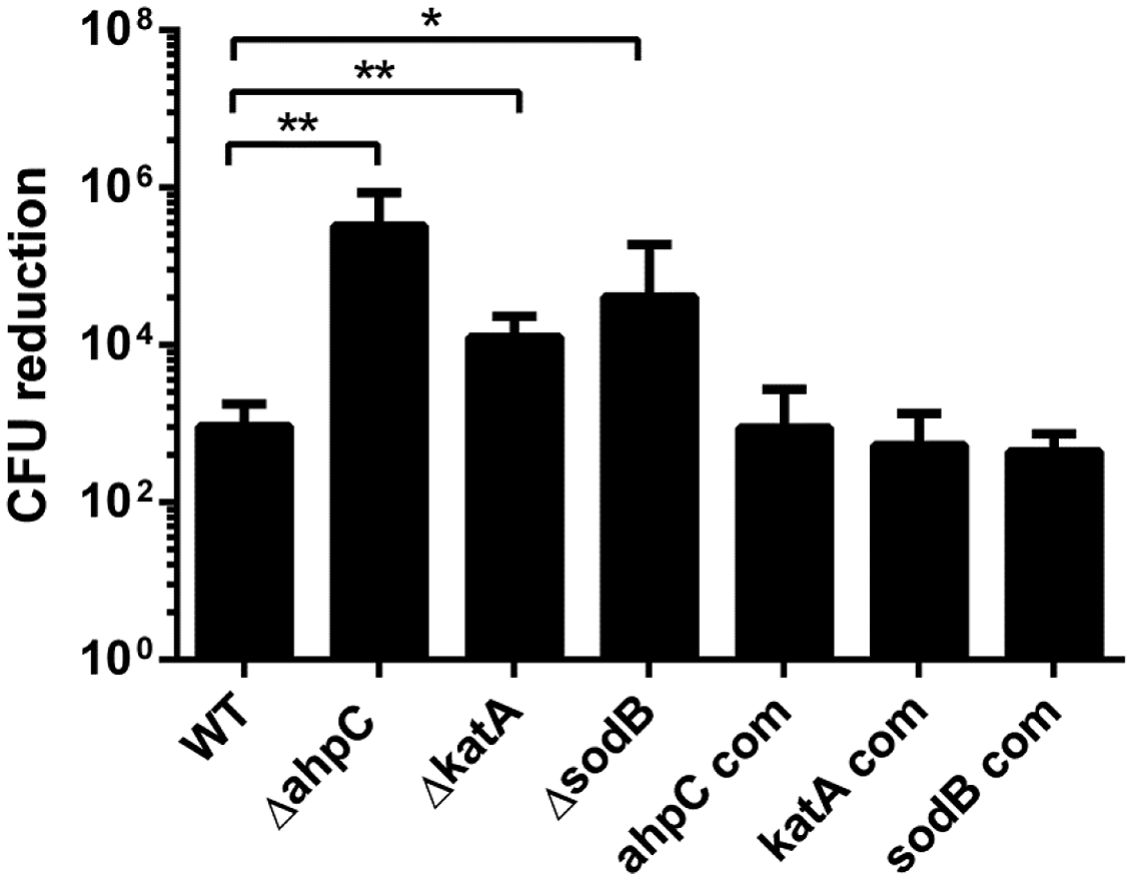
**Growth defects in the *ahpC, katA*, and *sodB* mutants under aerobic conditions**. The results show the means and SD of the levels of CFU reduction in triplicate samples after 12 h culture under aerobic conditions. The *ahpC* com, *katA* com, and *sodB* com are complementation strains of *ahpC*, *katA*, and *sodB*, respectively. The experiment was repeated three times and produced similar reduction patterns. The initial CFU levels were approximately 10^9^ CFU ml^-1^ in all the tested stains. Statistical analysis was conducted by using the Student’s *t*-test. *P≤ 0.05, **P< 0.01.

*Campylobacter jejuni* possesses only single gene copies of catalase (*katA*), alkyl hydroperoxide reductase (*ahpC*), and superoxide dismutase (*sodB*); they are primary enzymes for the detoxification of H_2_O_2_, organic peroxides, and superoxide, respectively. Since increased ROS accumulation reduced *C. jejuni* growth under oxygen-rich conditions (**Figure 1**), we investigated the contribution of these ROS-detoxification genes to *C. jejuni* survival under aerobic conditions. The growth of the *katA*, *ahpC*, and *sodB* mutants was significantly impaired under aerobic conditions, and the most substantial reduction was observed in the *ahpC* mutant (**Figure 2**). Complementation restored the aerotolerance of the mutants to the WT level (**Figure 2**), suggesting that the reduced aerotolerance in the mutants is directly related to their gene function in oxidative stress defense. A few studies have thus far shown the association of oxidative stress with the aerotolerance of *Campylobacter*. A mutation of *fdxA*, which is located upstream of *ahpC* and encodes the ferredoxin FdxA, affects the aerotolerance of *C. jejuni* (van Vliet et al., 2001). A double mutation of *bcp* and *tpx*, which encode the bacterioferritin comigratory protein (Bcp) and thiol peroxidase (Tpx), respectively, results in growth retardation under high aeration conditions (Atack et al., 2008). Baillon et al. (1999) reported that an *ahpC* mutation reduces aerotolerance in *C. jejuni* 81116. In this study, we showed that *ahpC* plays a more critical role than *katA* and *sodB* in the survival of *C. jejuni* under aerobic conditions (**Figure 2**). In other bacteria, antioxidant genes are differentially associated with aerotolerance depending on the bacterial species. A *katA* mutation increased the sensitivity of *Helicobacter hepaticus* to atmospheric oxygen (Belzer et al., 2011). In *Bacteroides fragilis*, mutations of oxidative stress defense genes, such as *ahpCF* and *katB*, rendered this obligate anaerobic bacterium more sensitive to aerobic stress than WT (Rocha et al., 1996). However, peroxide resistance genes, including *ahpC*, are not essential for aerotolerance in *Streptococcus pyogenes*, a catalase-negative facultative anaerobe (King et al., 2000).

A correlation between aerotolerance and the cellular levels of SOD has been reported; higher levels of SOD is produced in aerotolerant strains than oxygen-sensitive strains, and increased SOD production confers protection from high oxygen stress (Tally et al., 1977). The activity of SOD is essential for the aerotolerance of *Porphyromonas gingivalis*, an anaerobe implicated in chronic periodontitis, and mutations of SOD significantly reduced the viability of *P. gingivalis* in aerobic conditions (Nakayama, 1994). In this study, we also observed that a *sodB* mutation decreased aerotolerance in *C. jejuni* (**Figure 2**). However, a *sodB* mutation does not affect the growth of *Campylobacter coli* under aerobic conditions even with vigorous shaking at 150 rpm (Purdy et al., 1999). The differential impact of a *sodB* mutation on aerotolerance between *C. jejuni* and *C. coli* remains unexplained. However, the findings in this study suggest that the resistance to aerobic stress is different between *C. jejuni* and *C. coli*, although these two *Campylobacter* species are phylogenetically close to each other.

### Morphological Changes in the Oxidative Stress Defense Mutants under Aerobic Conditions

**FIGURE 3:**
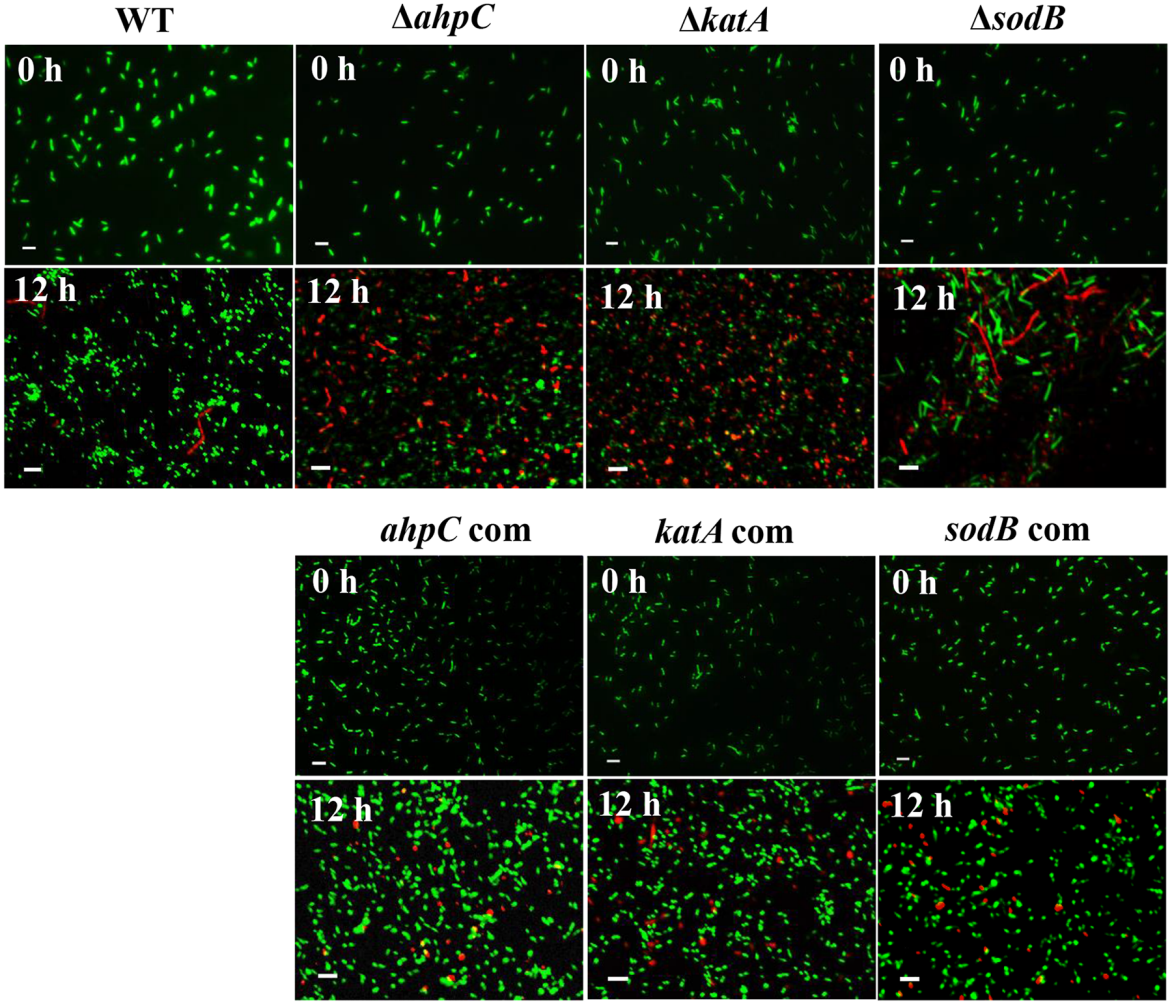
**Morphological changes in the oxidative stress defense mutants under aerobic conditions**. Samples were stained with the Live/Dead BacLight Bacterial Viability Kit (Life Technologies). Scale bar represents 2 μm. The images are representative of three independent experiments. The *ahpC* com, *katA* com, and *sodB* com are complementation strains of *ahpC*, *katA*, and *sodB*, respectively.

The viability of *C. jejuni* was alternatively observed with fluorescence microscopy after the LIVE/DEAD staining. Consistent with the viability reduction under aerobic conditions (**Figure 2**), the population of red (i.e., dead) cells markedly increased in the oxidative stress defense mutants under aerobic conditions, whereas green (i.e., live) cells constituted the major population in WT (**Figure 3**). The emergence of coccoidal cells was observed in WT, *ahpC*, and *katA* mutants with substantial dwarfing in size (**Figure 3**). Interestingly, in contrast, the *sodB* mutant exhibited significantly elongated cellular morphology (**Figure 3**). Despite similar levels of viability reductions between the *sodB* mutant and the *katA* mutant (**Figure 2**), their bacterial morphologies were completely different under aerobic conditions (**Figure 3**). Complementation increased the live cell population in the mutants and restored the morphology of the mutants similar to WT (**Figure 3**). These results exhibit that *C. jejuni* undergoes significant morphological changes under aerobic conditions and that the antioxidant genes are associated with *C. jejuni* morphology.

### Lipid and Protein Oxidation under Aerobic Conditions

**FIGURE 4:**
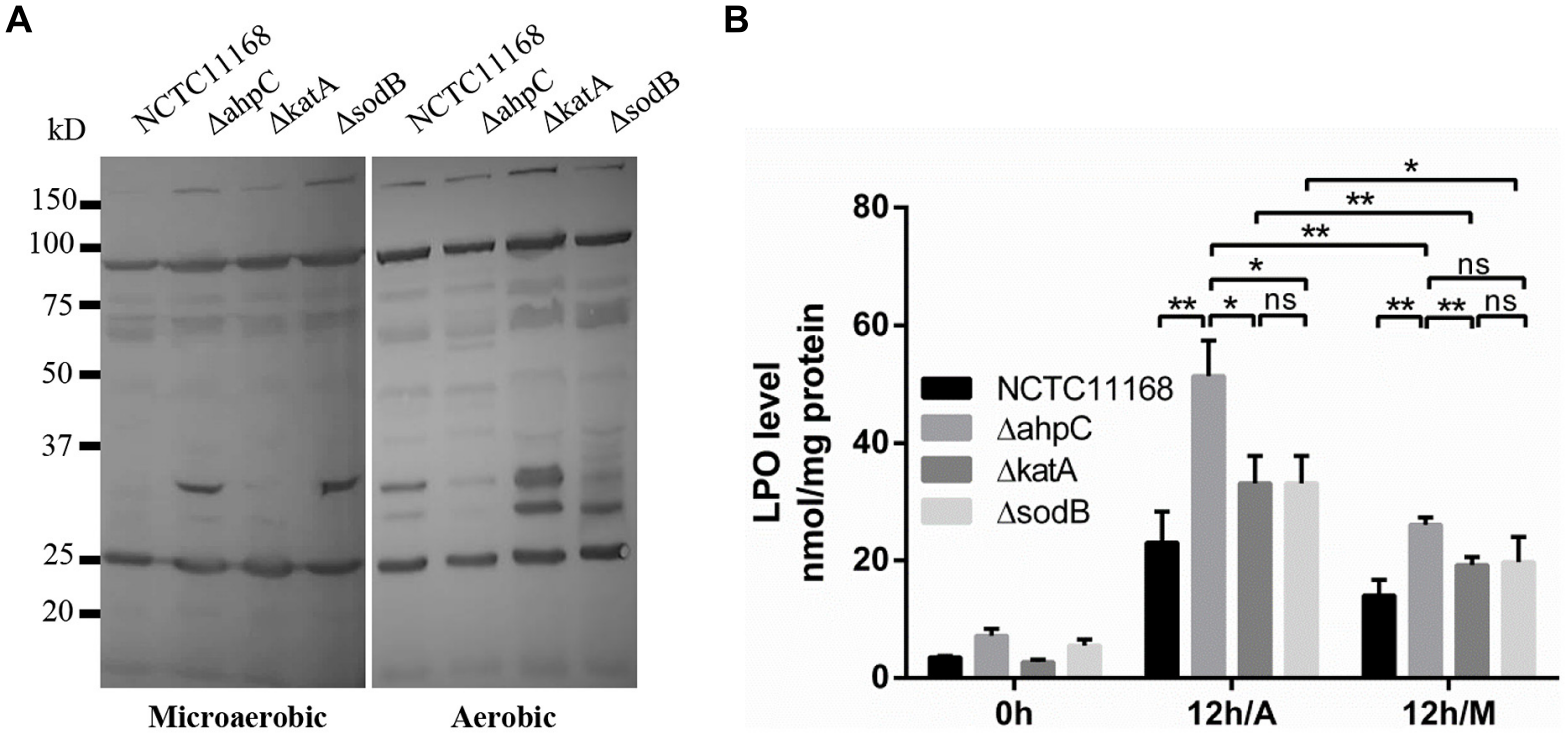
**Oxidation of proteins and lipids in the oxidative stress defense mutants under microaerobic and aerobic conditions**. **(A)** Protein oxidation was measured by western blotting with monoclonal antibodies that recognize protein carbonylation. **(B)** Lipid peroxide (LPO) levels were increased in the oxidative stress defense mutants under aerobic condition. The LPO levels were determined after 12 h culture under aerobic conditions (12 h/A) and 12 h under microaerobic conditions (12 h/M). Results shown are the means and SD of triplicate samples. The experiment was repeated three times and all results produced similar results. Statistical significance was determined by one-way ANOVA using GraphPad Prism 6. *P< 0.05, **P< 0.01, ns, *P* > 0.05.

Since ROS may damage macromolecular cellular components, such as fatty acids and proteins, we analyzed the levels of protein and lipid oxidation in the oxidative stress defense mutants under aerobic conditions. Protein carbonylation occurs as a result of protein oxidation; thus, the protein carbonylation level is indicative of protein damage by oxidative stress. Protein carbonylation was detected by using anti-DNP antibody. Increased levels of protein carbonylation were observed under aerobic conditions in both WT and the mutants (**Figure 4A**). Particularly, the *katA* mutant exhibited markedly increased protein carbonylation, compared to WT and the *ahpC* and *sodB* mutants (**Figure 4A**). Lipid peroxidation was also significantly increased in the mutants than WT under microaerobic and aerobic conditions (**Figure 4B**). The most substantial increase in lipid peroxidation was observed in the *ahpC* mutant (**Figure 4B**), signifying the role of *ahpC* in the detoxification of lipid peroxides in *C. jejuni*. Based on these results, the substantial viability reduction in the *ahpC* mutant under aerobic conditions (**Figure 2**) is most likely to result from lipid oxidation in *C. jejuni*.

### Emergence of Viable-But-Non-Culturable (VBNC) Cells under Aerobic Stress

Based on the morphological changes under aerobic conditions from spiral rods to coccoidal forms (**Figure 3**), we hypothesized that the emergence of coccoidal forms under aerobic conditions would be associated with oxidative stress. Exposure of *C. jejuni* to aerobic stress for 12 h significantly enhanced the formation of coccoid morphology, although *C. jejuni* is usually characterized by its typical helical rod shape. Counting of coccoid cells with fluorescence microscopy revealed that approximately 48.7% of *C. jejuni* cells established coccoid morphology after 12 h exposure to aerobic conditions, whereas only 2.6% of *C. jejuni* population turned to coccoidal forms under microaerobic conditions (**Table 2**). Interestingly, antioxidant treatment markedly reduced the population of coccoidal forms under aerobic conditions (**Table 2**), strongly indicating that oxidative stress is associated with the morphological changes. These results indicate that the formation of coccoidal cells was induced in *C. jejuni* under aerobic conditions through oxidative stress.

**Table 2:** Formation of coccoidal *Campylobacter jejuni* under different oxygen conditions.

Time	Microaerobic	Aerobic	Aerobic/antioxidant^†^
0 h	2.4 ± 0.35	1.9 ± 0.35	1.0 ± 0.30
4 h	4.9 ± 0.45	14.9 ± 2.19	1.3 ± 0.20
8 h	5.3 ± 0.82	27.3 ± 1.83**	4.6 ± 0.71
12 h	2.6 ± 0.42	48.6 ± 5.18^∗∗∗^	8.2 ± 0.46

*Percentage of coccoidal forms was obtained based on fluorescence microscopic observation, and the results show the mean and the SD of three different experiments.*

*^†^*N*-acetyl cysteine (1 nM) was used as an antioxidant.*

Statistical analysis was performed with the Student’s *t*-test by comparison between the microaerobic and aerobic samples. **P < 0.01, ^∗∗∗^*P*< 0.001.

**FIGURE 5:**
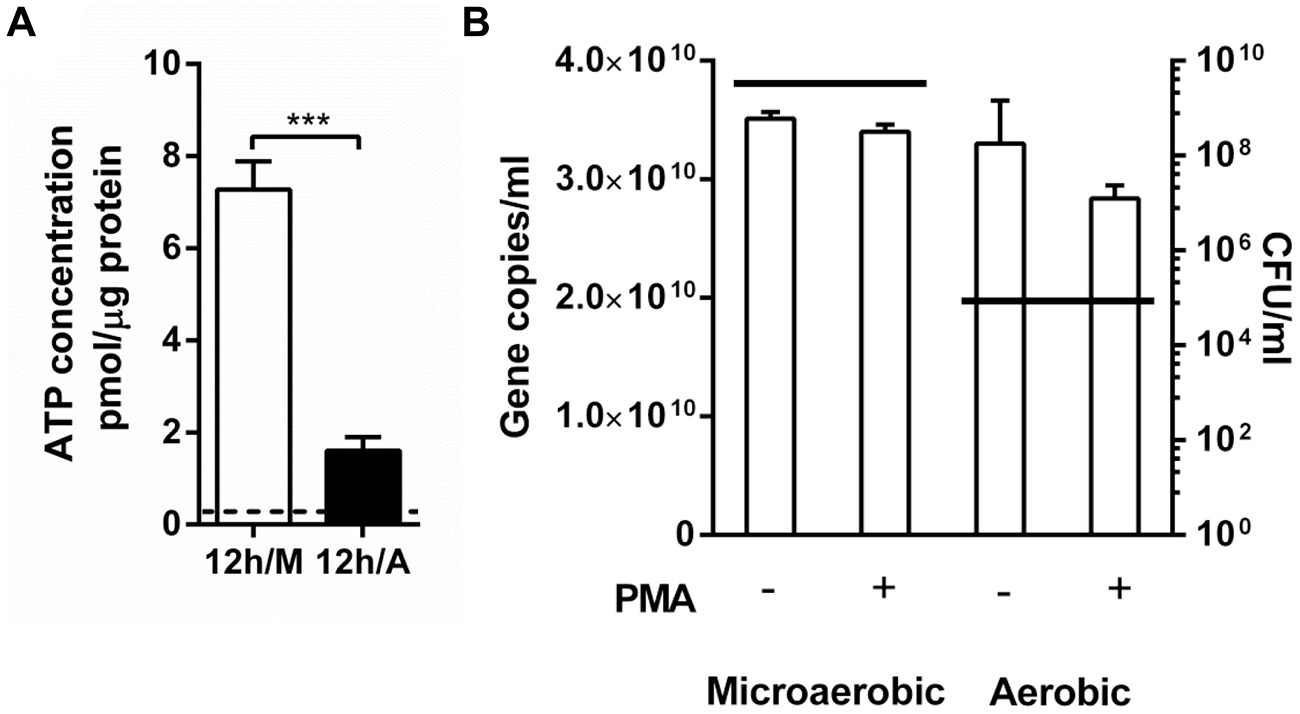
**Comparison of intracellular ATP levels and viability in *C. jejuni* between aerobic and microaerobic conditions. (A)** The concentrations of intracellular ATP in *C. jejuni* after 12 h culture under microaerobic conditions (12 h/M) and aerobic conditions (12 h/A). The initial ATP level at 0 h is indicated with a dotted line. A Student’s *t*-test was carried out for statistical analysis. ^∗∗∗^*P* < 0.001. **(B)** Quantitative PCR (qPCR) was performed with or without PMA treatment after 12 h growth under different oxygen conditions. A horizontal line indicates the average CFU levels of the samples that were used for qPCR.

Since the dwarfing morphology of *C. jejuni* under aerobic conditions is similar to that of VBNC cells, we hypothesized that aerobic conditions may trigger the physiological switch of *C. jejuni* to a VBNC state. Although cells in a VBNC state do not grow on normal laboratory culture media, they are still viable and exhibit decreased intracellular ATP levels (Tholozan et al., 1999). The physiological activity of *C. jejuni* was evaluated by measuring the intracellular ATP concentrations. After 12 h when the majority of *C. jejuni* was in coccoidal forms, the ATP levels were significantly decreased in aerobic conditions compared with microaerobic conditions (**Figure 5A**), suggesting that coccoidal *C. jejuni* is still alive but physiologically inactive. In addition, the presence of VBNC cells was also determined by using PMA–qPCR. PMA is a DNA-binding dye and impermeable to the membrane of live cells. PMA modification of DNA by photolysis renders DNA non-amplifiable by PCR. Thus, PMA is often used to quantify DNA between live and dead cells (Nocker et al., 2006) and also to determine the levels of VBNC population (Dinu and Bach, 2013). The qPCR results from samples without PMA treatment reflect the population of whole cells, both live and dead, whereas PMA-treated sample shows only the population levels of live cells including VBNC cells. Based on gene copy numbers, there was a reduction in *C. jejuni* population in the PMA-treated samples compared to the non-PMA-treated samples after 12 h culture under aerobic conditions; however, the difference was not statistically significant (**Figure 5B**). This observation suggests that *C. jejuni* viability was not decreased significantly despite the substantial CFU reduction under aerobic conditions (**Figures 2** and **5B**), indicating that *C. jejuni* mostly lost its culturability, not viability, under the aerobic conditions. These findings strongly suggest that aerobic exposure induces the formation of VBNC cells in *C. jejuni*.

Bacteria in a VBNC state typically develop coccoid morphology by exposure to certain stress conditions. Despite being still alive, cells in a VBNC state do not grow on laboratory media and exhibit reduced energy consumption (Oliver, 2010). Upon removal of the stress, however, VBNC cells return to their original morphological shape and regain their physiological conditions and culturability. Therefore, the entry into a VBNC state is considered as an important strategy for bacteria to survive under stress conditions (Oliver, 2010). Many pathogenic bacteria, including *C. jejuni*, are known to form VBNC cells (Oliver, 2010). Importantly, pathogenic bacteria still maintain their infectivity in a VBNC state, because the resuscitation of VBNC cells enables pathogens to initiate an infection process (Oliver, 2010). *C. jejuni* cells in a VBNC state recover their ability to adhere to and invade human intestinal cells (Chaisowwong et al., 2012; Patrone et al., 2013) and to colonize animal intestines (Baffone et al., 2006). Multiple factors have been reported to induce a VBNC state in *Campylobacter*, including prolonged incubation at cold temperature (Chaisowwong et al., 2012), starvation (Klancnik et al., 2009), acid stress (e.g., formic acid at pH 4; Chaveerach et al., 2003), and polyphosphate kinase 1(PPK1; Gangaiah et al., 2009). In addition, our recent findings and previous studies of others have consistently shown the appearance of coccoidal forms in *C. jejuni* under aerobic conditions (Klancnik et al., 2013; Kim et al., 2015). In this study, we showed that aerobic culture increased the population of VBNC cells, whereas antioxidant treatment reduced the formation of VBNC cells (**Table 2**). These findings strongly indicate that oxidative stress induces a VBNC state in *C. jejuni*. Similarly, the impact of oxidative stress on the induction of a VBNC state has also been reported in *Vibrio* species. An *oxyR* mutant of *Vibrio vulnificus*, which is defective in catalase activity, is more likely to enter a VBNC state by cold temperatures compared to the parent strain, and the catalase activity has a direct correlation with culturability (Kong et al., 2004). Supplementation of culture media with H_2_O_2_-degrading compounds, such as catalase, enhances the resuscitation of VBNC cells in *Vibrio parahaemolyticus* (Mizunoe et al., 2000). By extrapolation from the reports in *Vibrio*, when *C. jejuni* is exposed to oxygen-rich conditions, this microaerophilic pathogenic bacterium may switch its physiological state to a VBNC form. In summary, the findings in this study demonstrate the impact of oxidative stress defense on *C. jejuni* growth and morphology under aerobic conditions. We still await further investigations to explain details about the molecular mechanisms for the formation of VBNC cells in *C. jejuni* under oxygen-rich conditions. Additionally, we performed the experiment in this study at 42°C with changes only in oxygen conditions (i.e., microaerobic vs. aerobic) to define the impact of aerobic stress on *C. jejuni* physiology. However, in the environment and poultry meat, *C. jejuni* would be likely to be exposed to aerobic conditions at lower temperatures than 42°C. In view of this, it would be an interesting future study to investigate the role of oxidative stress defense in *C. jejuni* survival under aerobic conditions at low temperatures in food or water settings.

## Conflict of Interest Statement

The authors declare that the research was conducted in the absence of any commercial or financial relationships that could be construed as a potential conflict of interest.
